# A framework for privacy-preserving similarity search of massive multi-party genomic data

**DOI:** 10.3389/fpls.2025.1684243

**Published:** 2025-12-03

**Authors:** Jia Liu, Yanping Xu, Abdullah Gera, Xiaoning Li, Liping Zhao, Xiaoli Zhu

**Affiliations:** 1 Office of Academic Research, Weifang Institute of Technology, Weifang, China; 2 Office of Academic Research, Nilai University, Nilai, Negeri Sembilan, Malaysia

**Keywords:** privacy protection, federated learning, genomic selection, locality-sensitive hashing, plant breeding

## Abstract

To address the challenges of data silos across different institutions, privacy concerns, and the multi-party genomic data matching problem in crop breeding, this paper proposes a Fed-LSH framework. This framework is a collaborative framework integrating privacy- enhanced Locality-Sensitive Hashing (LSH) algorithm with Federated Learning. It enables participants can conduct cross- institutional similar genomic association analysis and elite allele identification, And they do not need to share raw genomic data with each other. This framework utilizes distributed hash index construction, outsourced computation, and encrypted similarity search to accomplish this task. Experiments show that Fed-LSH can achieve a hit rate of 60.72% ± 1.2% when recommending 4 candidates, using a 40×3 hash size on 3072-dimensional data (implemented on standard personal computers). It can select 4 candidates from 10,000 genomic fragments (each 3072-dimensional) in less than 0.5 seconds. These performance metrics indicate that Fed-LSH provides foundational technical support for privacy-preserving collaborative tomato breeding.

## 1 Introduction

The similarity matching technology of crop genes is one of the core technologies supporting modern agricultural genomics and crop improvement (Gao, 2021). Gene sequence similarity matching technology can help reveal the intrinsic association mechanism between genetic diversity, structural variation and crop traits by analyzing the genetic similarity among different crops and different varieties of the same crop, and then select suitable breeding plants, providing technical and theoretical support for precision molecular breeding and precision agriculture of crops (Rasmussen, 2020). However, genomic data is often huge in volume, and it is frequently confronted with the problem of high computational complexity. In cases where the length of the genome approaches millions of base pairs, the existence of polyploid genomes also makes the matching of similar genes in crops more complex. All these have put forward requirements and challenges for the rapid detection of massive gene data in crops (Y. Chen et al., 2020).

Rapid similarity matching of massive gene sequences is a key technology in modern crop genomics and genetic research, which can significantly enhance the efficiency of breeding. Currently, various efficient and fast computing models exist, including high-throughput SNP chips (Ganal et al., 2012), k-mer counting algorithms (Mahmood et al., 2012; Wilbur and Lipman, 1983), parallel similarity matching algorithms, and improved fast sequence alignment tools (Yonia et al., 2024). With the development of high-throughput sequencing technology and the establishment of massive genomic databases, numerous efficient algorithms have emerged, which can support the rapid comparison and similarity analysis of tens of thousands of genes (Hahn et al., 2024). One of the key improvements of these algorithms is the ability to complete the rapid similarity matching of large-scale gene data within a short time. For example, in the application of high- throughput SNP chips, large-scale gene similarity assessment and gene mapping in crop sample databases can be completed through the rapid genotyping of thousands or even tens of thousands of markers across the entire genome. In the application of k-mer counting algorithms, through the extraction of short sequence gene features, comprehensive matching and acceleration of large-scale protein/nucleic acid gene sequences can be achieved (such as the afree tool), which can significantly improve the comprehensive retrieval efficiency of homologous genes. The rapid gene matching based on k-mer counting algorithms is applicable to the matching of similar gene fragments in complex genomic environments. The new generation of DNA pattern matching algorithms, such as EPAPM and EFLPM (Ibrahim et al., 2023)enhance matching speed through parallel processing technology and improve similarity matching performance by providing error tolerance mechanisms, enabling the rapid completion of gene sequence similarity matching in complex and massive genomic similarity matching environments at the expense of certain accuracy.

In fact, multi-party gene matching, especially that among different regions and countries, has demonstrated multiple values in crop genetic breeding and trait improvement (Tao et al., 2019). Due to the differences in environmental adaptability and stress resistance of multi-party gene matching, it can significantly promote the genetic diversity of crop genetic breeding, the expression of heterosis, and enhance stress resistance, etc. At the same time, it can provide a rich genetic resource pool for coping with different climates and ecological environments, and support complex gene matching and genetic breeding (Snowdon et al., 2021; Jeynes-Cupper and Catoni, 2023; Legarra et al., 2023) However, important germplasm resources globally are scattered among different institutions (such as the Consultative Group on International Agricultural Research (CGIAR), national gene banks, and private breeding companies). Adopting multi-party gene matching would face the problem of local genomic data leakage. Due to competition and privacy concerns, original data is often not directly shared (Harmanci and Gerstein, 2016). The core concept of federated learning technology is distributed machine learning, which allows multiple parties to collaboratively train models without sharing data. It is a solution to this problem (Li et al., 2023). Through federated learning technology, each institution retains the original genomic data and only exchanges encrypted model parameters (gradients or hash indexes).

Traditional centralized databases, such as NCBI (Schoch et al., 2020) and SRA (Katz et al., 2022), require public data uploads, which cannot meet the privacy protection needs of institutions. Traditional encrypted retrieval methods [such as homomorphic encryption BLAST (Rupa et al., 2023)] have huge computational costs (retrieval of TB-scale data requires response times on the order of hours), making them difficult to be practically applied. Federated learning (FL) is a potential solution to this problem. However, although FL algorithms support distributed training, they lack corresponding algorithm and application framework support, especially for distributed algorithms for rapid and massive matching of similar crop genes in a multi-party privacy protection environment (Wu et al., 2025).

The Locally Sensitive Hash (LSH) algorithm, as an efficient approximate search tool in gene matching (Baker and Langmead, 2023), works by mapping similar gene sequences into the same hash bucket, thereby significantly accelerating large-scale searches by reducing the number of exact sequence comparisons (K. Chen and Shao, 2023). LSH supports various similarity measures such as Euclidean distance, Jaccard similarity, and Hamming distance, and can be adapted to different data types by designing corresponding hash function families (Martínez et al., 2022), demonstrating multi-metric adaptability. For instance, some improved LSH-based algorithms can achieve performance acceleration by several orders of magnitude while maintaining a recall rate of over 0.95 and keeping resource consumption low (Jia et al., 2021; Satuluri and Parthasarathy, 2012; Sundaram et al., 2013; Yu et al., 2017, 2019), thus offering significant performance advantages. For example, the LSH-ALL-PAIRS algorithm demonstrates high sensitivity in gene detection, capable of identifying local sequence similarities as low as 63% in massive genomic datasets of tens of megabases (Buhler, 2001). The core advantage of LSH in the field of gene similarity matching lies in its ability to perform rapid similarity matching detection on ultra-large-scale gene data (Buhler, 2001). Moreover, LSH is often combined with other algorithms such as deep learning and generative models, expanding the complexity of biological gene analysis dimensions (Lall et al., 2022; Tavakoli, 2019). Therefore, LSH provides high application and research value in offering efficient and scalable approximate search capabilities for massive gene data, integrating similar gene sequence comparisons, maintaining algorithm sensitivity and accuracy, and integrating with other feature extraction algorithms.

To break through the above-mentioned bottlenecks, this paper proposes the Fed-LSH framework (Federated Locality-Sensitive Hashing), which deeply integrates Locality-Sensitive Hashing (LSH) with federated learning to achieve privacy-protected similarity search for tomato genomic data. The innovation of this framework lies in its ability to conduct similarity search without sharing local raw data through the improved Fed-LSH framework. Based on the data, rapid similarity matching is carried out. Meanwhile, cloud-assisted machine learning technology is adopted for batch rapid local gene operations, reducing the local computing burden and meeting the needs of multiple institutions for rapid similarity gene matching.

## 2 Method design

### 2.1 Federated LSH architecture

Cloud Server: The cloud server maintains the consistency of the global projection matrix of the LSH algorithm, generates the LSH algorithm projection matrix w, and transmits it to each institution (client) to complete the cloud-assisted machine learning of the LSH algorithm. Through the data  x' and w' transmitted by the local node, the encrypted HashTable' is generated.

Clients: Each institution (Client) holds private genomic data, generates encryption keys, encrypts local data x and projection matrix w to form x' and w'. Meanwhile, based on the HashTable' returned by the cloud server, it verifies the correctness of HashTable' and generates the real HashTable.

Data Server: Data server aggregates the global hash table and responds to cross-node similarity queries.

**Figure 1:**
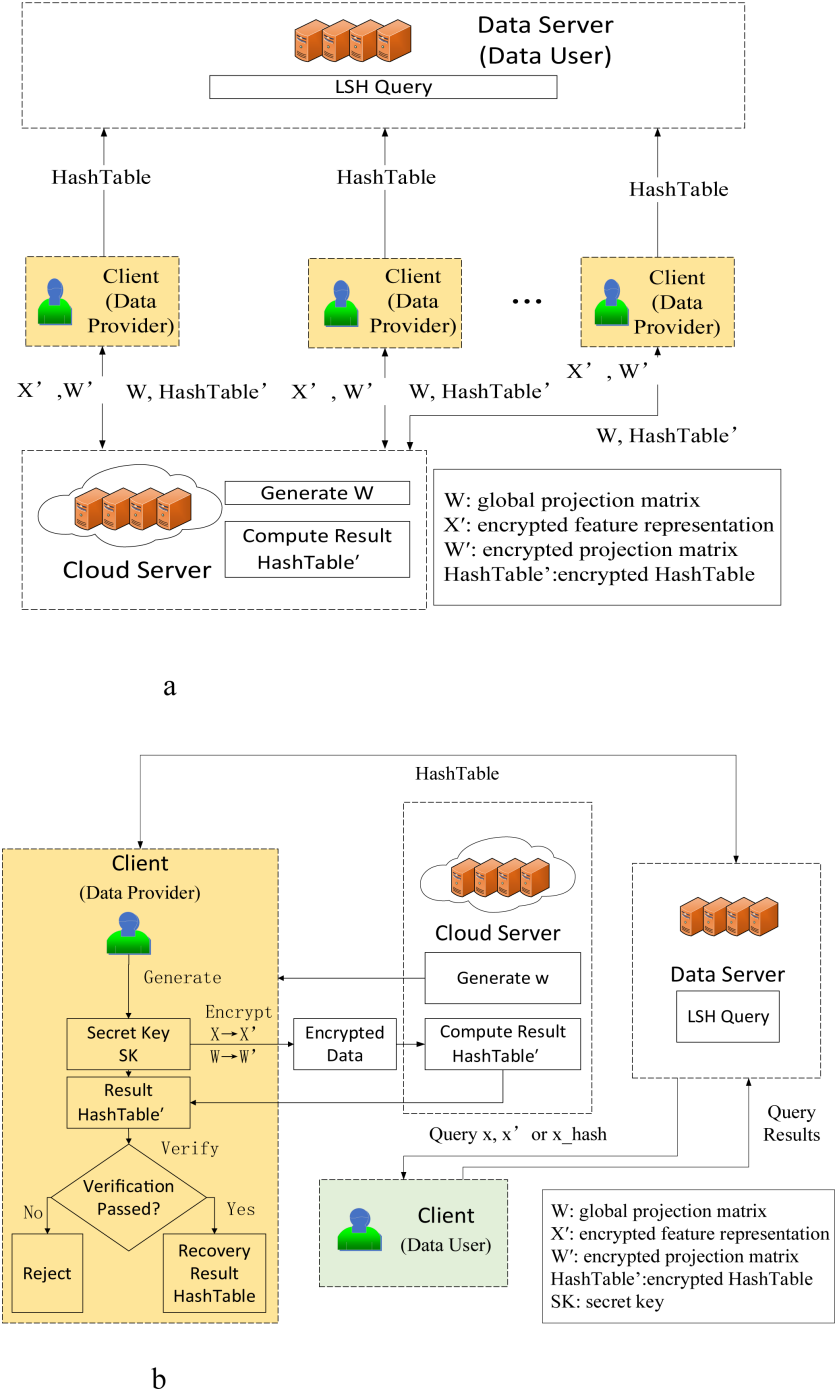
**(A)** System architecture overview. **(B)** Operation flow and data exchange protocol.

**Figure 2:**
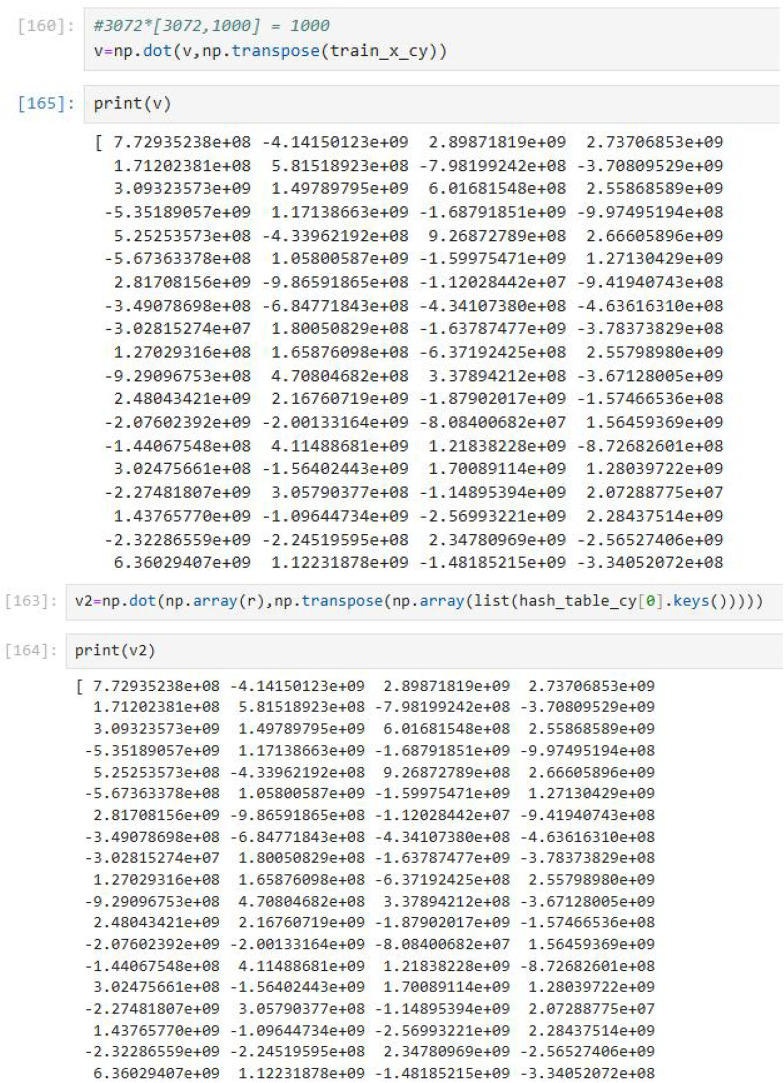
Python experimental results of the verification algorithm.

Clients can generate HashTables locally using the projection matrix generated by the Cloud Server from their stored private genetic data and send it to the Data Server. Alternatively, they can use the proposed cloud-assisted machine learning technology to outsource part of the LSH algorithm’s computations to the Cloud Server, which will batch-generate encrypted HashTable', and return it to the Clients. The Clients then decrypt the batch HashTable' to obtain the original HashTable and send it to the Data Server. For the specific architecture, please refer to **Figures 1**, **2**.

### 2.2 Framework operation steps

#### 2.2.1 Global projection matrix generation

The Cloud Server generates a global projection matrix W, where $W\in {\mathbb{R}}^{t\times d}$, and transmits W to each Client. t is the number of rows of the global projection matrix W, the size of the hash. The larger the t, the higher the hash discrimination degree, which can better distinguish similar and dissimilar data. A shorter hash size improves the query speed, a longer hash size improves the accuracy rate, but the computational cost increases. d represents the feature dimension of the original data, the larger the d, the higher the data dimension, and the more complex the represented data is.

#### 2.2.2 Local key generation

Each institution (Client) acts as a data provider holding private data. Client generates its own key. The data of each Client is $X\in {\mathbb{R}}^{m\times d}$. With the parameters λ=(m,d), the Client selects 2l Givens matrices. $P_{1},P_{2},\cdots P_{l},Q_{1},Q_{2},\cdots Q_{l}$ and a random real number α as the secret key $SK=\left\{\alpha ,P_{1},P_{2},\cdots P_{l},Q_{1},Q_{2},\cdots Q_{l}\right\}$.

The Givens matrix is also known as the elementary rotation matrix, as shown in Equation 1. It is a sparse matrix with most of its elements being 0 and is orthogonal, which can reduce the computational load in matrix multiplication. The client randomly selects an integer θ∈(0,2*π), and constructs the Givens matrix Tij as follows:

- Let c=cosθ, s=sinθ.
- Set the i-th and j-th main diagonal elements to c.
- Set the other diagonal elements to 1.
- Set the element at the i-th row and j-th column to s.
- Set the element at the j-th row and i-th column to −s.

Set all other elements to 0.

(1) $$T\left(i,j\right)=\left[\begin{array}{cc} \begin{array}{c} 1\\ \ddots \\ 1 \end{array}\\ \begin{array}{cc} c\\ 1\\ \vdots & \ddots \end{array} & \begin{array}{c} s \end{array}\\ \begin{array}{c} -s\\ \vdots \end{array} & \begin{array}{c} 1\\ c\\ \begin{array}{c} 1\\ \ddots \\ 1 \end{array} \end{array} \end{array}\right]$$

#### 2.2.3 Local data encryption

Client encrypts the data into $X^{\prime }$ and $W^{\prime }$ using SK, as shown in Equations 2, 3.

(2) $$X^{\prime }=P_{1}P_{2}\cdots P_{l}\left(\alpha X\right)Q_{1}Q_{2}\cdots Q_{l}$$

(3) $$W^{\prime }=\left(\alpha W\right)Q_{1}Q_{2}\cdots Q_{l}$$

#### 2.2.4 Cloud-assisted machine learning

The Cloud Server computes the encrypted hash code and sends the encrypted hash table $HashTable^{'}$ back to the Client, as shown in Equation 4.

(4) $$HashTable^{'}=W^{\prime }{X^{\prime }}^{T},\;W\in {\mathbb{R}}^{t\times d}$$

The Client verifies the correctness of the encrypted hash table $HashTable^{'}$. The Client randomly selects a vector $r\in {\mathbb{R}}^{1\times t}$ to calculate $V_{1}$ and $V_{2}$, as shown in Equations 5, 6. If the value of $V_{1}$ and $V_{2}$ are equal, then the $HashTable^{'}$′ is correct.

(5) $$V_{1}=(rW^{\prime }){X^{\prime }}^{T}$$

(6) $$V_{2}=rHashTable^{'}$$

#### 2.2.5 Restore the original hash table

The Client restores the HashTable, as shown in Equation 7.

(7) $$HashTable=\frac{1}{\alpha^{2}}(HashTable^{'}P_{1}P_{2}\cdots P_{l})$$

#### 2.2.6 Send the hash table to the data server

The Client will either use cloud-assisted machine learning to batch-generate or directly adopt the W−computed HashTable and send it to Data server.

#### 2.2.7 Fast query of multiple similar gene data

If the genetic data does not require privacy protection, the Client can choose to send the raw data to the Data Server. The Data Server then computes $WX^{T}$ to form the hash value of the query data, enabling a fast LSH query on the Data Server side.

For a small amount of genetic data that requires privacy protection, the Client can calculate $WX^{T}$ and send the hash value of the data to the Data Server for similarity queries.

To conduct batch queries of similar data, the Client can leverage the Cloud Server to generate a cloud-assisted batch query data HashTable. The Cloud Server generates $HashTable^{'}$ through the privacy-protected cloud-assisted machine learning algorithm proposed above. The Client then verifies and restores the original HashTable and sends it to the Data Server for rapid LSH-based similar data queries.

The Data Server generates the LSH algorithm hash table LshHashTable based on Equation 8 and the transmitted HashTable, and simultaneously selects one of the distance algorithms such as Euclidean distance, cosine distance, or L1 norm distance for the LSH algorithm similarity query, returning num_results results.

(8) $$\text{LshHashTable}==\left[\frac{HashTable+b}{num\_hash}\right]$$

## 3 Performance verification

### 3.1 Correctness verification

We denote P and Q as $P=P_{1}P_{2}\cdots P_{l}$, $Q=Q_{1}Q_{2}\cdots Q_{l}$ respectively. Each Client recovers the $H\text{ashTable from }H{\text{ashTable}}^{'}$ through $HashTable=\frac{1}{\alpha^{2}}(HashTable^{'}P)$, as shown in Equation 9.

(9) $$\begin{array}{l} HashTable=\frac{1}{\alpha^{2}}(HashTable^{'}P)\\ =\frac{1}{\alpha^{2}}\left(W^{\prime }{X^{\prime }}^{T}P\right)\\ =\frac{1}{\alpha^{2}}\left(\left(\alpha W\right)Q{\left(P\left(\alpha X\right)Q\right)}^{T}P\right)\\ =\frac{1}{\alpha^{2}}\left(\left(\alpha W\right)QQ^{T}{\left(\alpha X\right)}^{T}P^{T}P\right)\\ =\frac{1}{\alpha^{2}}\left(\alpha^{2}WX^{T}\right)\\ =WX^{T} \end{array}$$

Therefore, the recovered HashTable is the output of the original computation. Meanwhile, we prove the correctness of the verification algorithm. The Client verifies the correctness of the value computed by the Cloud Server by checking if $V_{1}=V_{2}$.

(10) $$\begin{array}{l} V_{1}=(rW^{\prime }){X^{\prime }}^{T}\\ =rW^{\prime }{X^{\prime }}^{T}\\ =rHashTable^{'}\\ =V_{2} \end{array}$$

Therefore, if $V_{1}=V_{2}$, the result is correct. Meanwhile, the code result implemented through Python code is as follows, which also proves the correctness of the verification algorithm, as shown in Equation 10.

### 3.2 Security verification

The CIFAR-10 dataset was used to perform the encryption operation of the Client-side key. We tested the blinding effect of the original data X multiplied by different numbers of $l\text{ Givens matrices},{\text{namely P}}_{1}{\text{P}}_{2}\cdots {\text{P}}_{l}\text{X}$. It can be seen that the Givens matrix has a good blinding effect on the original data, as shown in **Table 1**.

**Table 1:** Blinding effects of givens matrices with different **l** values on image data.

Cell Type Characteristic	T-bet^*^ hTregs	GATA3^*^ Tregs	RORyt^*^ Tregs
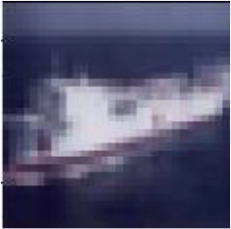	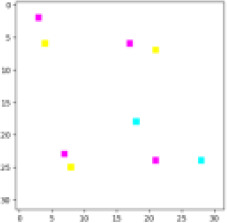	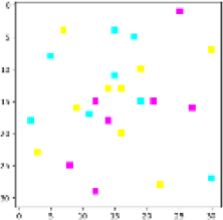	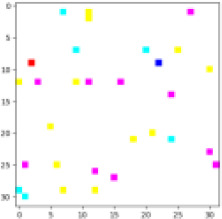
	51	61	71
	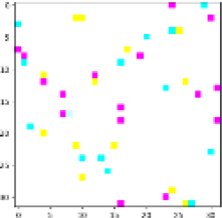	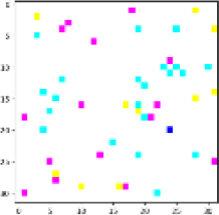	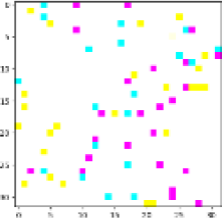

#### 3.2.1 The proposed algorithm has input/output privacy

In the proposed scheme, the original data of the Client, i. e., X, is hidden from the Data Server and the Cloud Server. In our scheme, the Client transforms X into X' by multiplying a real number with a series of basic rotation matrices. The selected Givens matrices are randomly generated. These basic rotation matrices are randomly chosen. The $P_{1}P_{2}\cdots P_{l}\text{ in the key }SK$ is only used locally and will not be transmitted over the network. Therefore, for the Cloud Server, if it needs to recover the original X, it has to guess the values of $P_{1}P_{2}\cdots P_{l}$. Assuming the probability of successfully guessing an elementary rotation matrix is $\frac{1}{\delta }$. Then the probability of successfully guessing l elementary rotation matrices is $negli\left(l\right)=\frac{1}{\delta^{l}}$, which is a non-polynomial time function.

In fact, since the values in the elementary rotation matrix are real numbers with a very wide range, the probability of successful guessing is extremely low. If one needs to guess the value of X, the probability of correctly guessing X is $negli\left(md\right)=\frac{1}{\delta^{md}}$. For the Cloud Server, the probability of successfully guessing the correct HashTable is $negli\left(tm\right)=\frac{1}{\delta^{tm}}$. All of these are non-polynomial time. Therefore, the probability that Cloud Server successfully guesses the series of Givens matrices, X and HashTable is relatively low. Similarly, for Data Server, since the Client only transmits HashTable to Data Server, the original data X is hidden from Data Server.

#### 3.2.2 The efficiency of the proposed algorithm on the client side is ((2md+2td+2tm+4ml+4dl)/(tdm)) efficient

We define scalar multiplication as SM and ignore linear computations such as scalar addition. For a Client, it performs md+td+2ml+4dl SM. During the encryption phase on the Client side, the Client executes md+td+2ml+4dl SM. The Cloud Server performs tdm SM in the computation phase. The Client recovers the HashTable by performing tm+2ml SM. Therefore, a single Client performs a total of 2md+2td+2tm+4ml+4dl SM. The Cloud Server performs tdm SM in total. The computational cost of the algorithm without cloud-assisted machine learning for the Client is mtd SM, which is as high as the computational complexity of the LSH algorithm using cloud assistance. Thus, the efficiency of the proposed method is ((2md+2td+2tm+4ml+4dl)/(tdm)).

## 4 Experimental verification and evaluation

### 4.1 Algorithm verification on CIFAR-10 dataset

- The verification experiment was conducted on a regular computer with a 2.5GHz CPU and 16GB of memory running the Ubuntu 22.04 operating system. Python 3.11 was used as the programming language. To verify the correctness of the algorithm, the CIFAR-10 image dataset was employed for simulation. To test the algorithm’s performance on crop gene data, the NCBI tomato gene dataset was utilized for application and performance evaluation.
- The CIFAR-10 dataset contains 60,000 image data across 10 categories. Each category consists of 6,000 32x32 images.
- The NCBI tomato gene data contains 5,156 tomato gene records.

**Figure 3:**
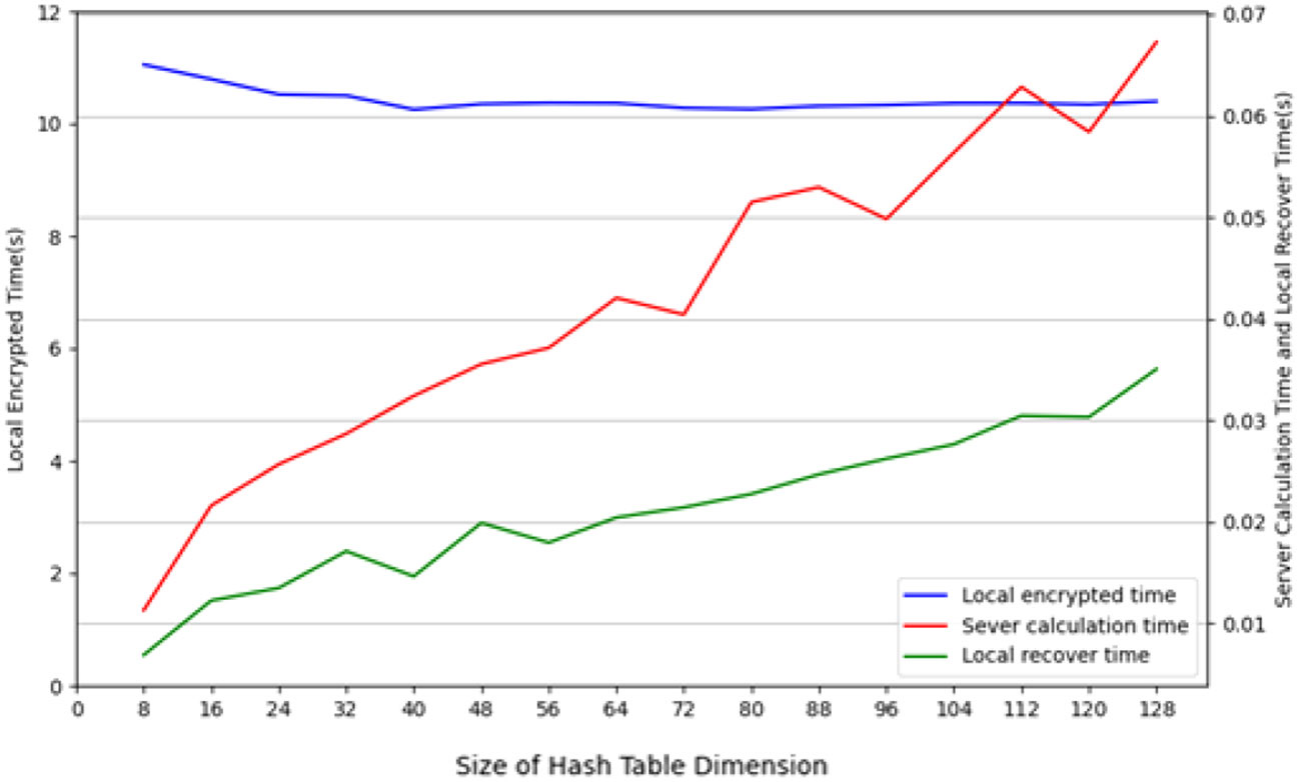
The running time of different-sized hash table on the client and cloud server on CIFAR-10 dataset.

**Figure 4:**
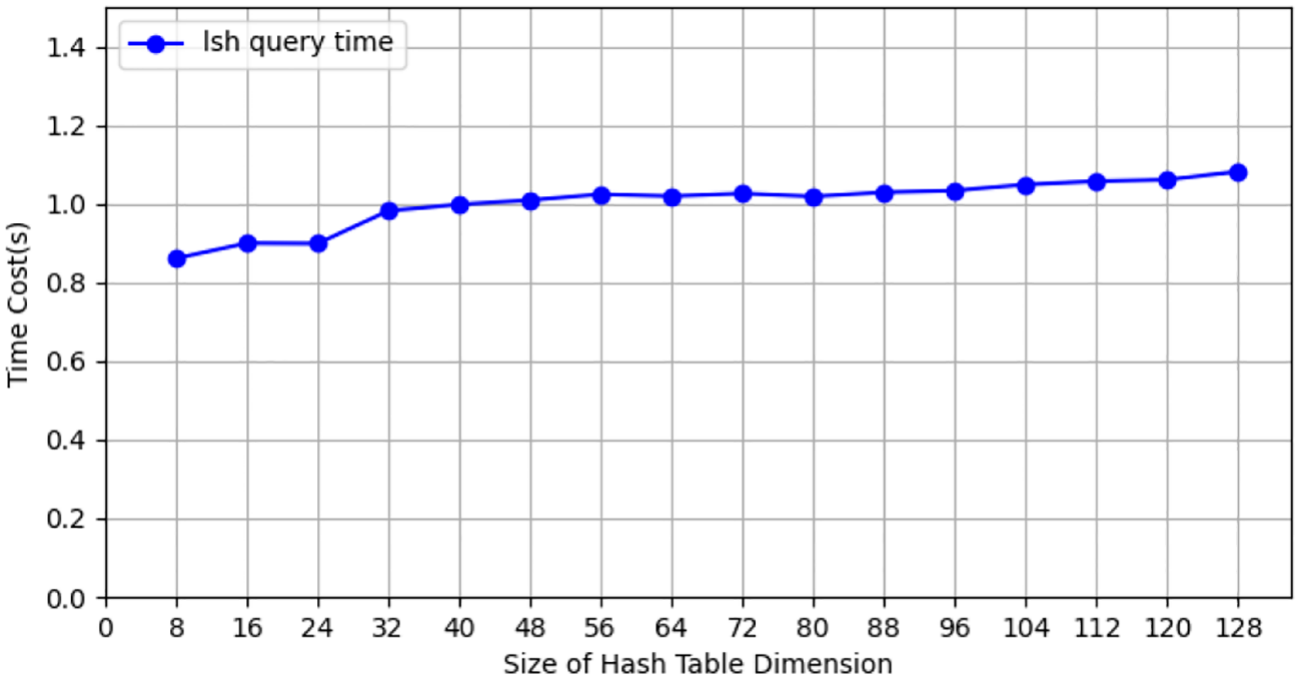
The query efficiency of data server for hash tables of different sizes on CIFAR-10 dataset.

**Figure 5:**
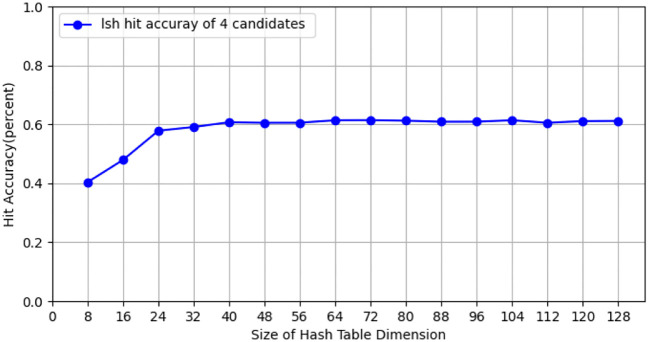
The hit rate of providing four candidate members for hash tables of different sizes on CIFAR-10 dataset.

During the testing process using the CIFAR-10 dataset, we conducted tests with the CIFAR-10 training dataset (50, 000 images), employing 10 client nodes for federated learning LSH algorithm testing, with each client containing 5,000 images. Performance tests were carried out on both the client and Cloud Server ends using different hash sizes. All experiments were repeated 10 times with different random seeds, and results were averaged for final reporting. The specific results are shown in **Figure 3**. The query efficiency at the Data Server end is presented in **Figure 4**, and the performance of hit rate providing 4 candidate results is shown in **Figure 5**.

Fed-LSH achieves a hit rate of 60.72 ± 1.2% (averaged over 10 runs) on CIFAR-10 dataset.

### 4.2 Performance evaluation on NCBI tomato dataset

We applied it on the tomato gene data from NCBI. When processing the tomato gene data from NCBI, due to the inconsistent lengths of the gene data, we performed slicing of the gene data. The specific algorithm is **Algorithm 1**.

Algorithm 1 **Gene data slicing and k-mer extraction for genomic hashing.**


• Numericize the NCBI tomato gene data as follows: {'A': 0, 'T': 1, 'C': 2, 'G': 3}.
• Set the sequence length sequence_length and the overlap for the gene data slices.
• For each gene data, slice the data based on sequence_length and overlap.
• For each kmer slice, if there is a part that is shorter than sequence_length, fill it with -1.
• Encapsulate each kmer of the slice and the corresponding count-th gene data to form a (kmer, count) pair.
• For each gene data, a total of n_chunks pairs of (kmer, count) are obtained.
• Gene data slicing and k-mer extraction for genomic hashing.

With a sequence length of 3072 and an overlap of 100, after processing the gene data, we obtained a total of 298,613 gene slice data.

To prevent data duplication, in practical applications, a unique identifier (UUID) can be assigned to each gene fragment. During the process of building the hash index, UUID conflict detection is carried out first. Once duplicate gene items are detected, the system can skip them. This ensures that the hash index only contains unique gene sequences and avoids duplicate analyses.

**Figure 6:**
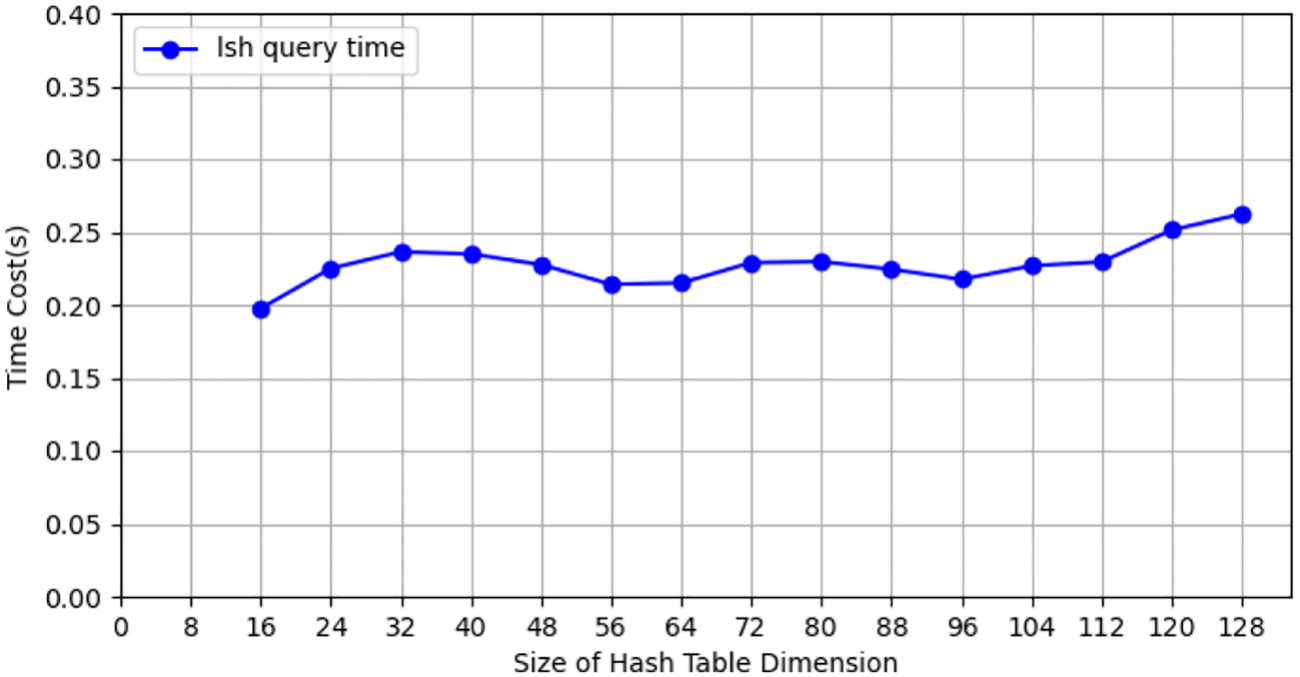
The query efficiency of providing four candidate members from different Hash Tables of NCBI tomato gene data.

We selected 10,000 gene data records for performance testing. Each client had 1,000 gene data records. Four candidate results were selected to test the performance of multi-party queries. The specific performance is shown in **Figure 6**. From the performance test, it can be seen that the performance of similar gene queries is relatively high. On an ordinary computer without GPU acceleration, the time required to select four candidate sets from 10,000 gene fragments of 3072 in length is less than 0.5 seconds.

**Tables 2**–**4** compares the performance of the Proposed Fed-LSH with other privacy-preserving methods (HELib, DP, SMPC) in terms of computational cost, query latency, and privacy level. The Proposed Fed-LSH achieves low computational cost and low query latency (<0.5s) while maintaining a high privacy level. In contrast, HELib has very high computational cost and long query latency (>3.6s) but high privacy; DP offers low computational cost and low latency (2.67s) but only low privacy; SMPC has high computational cost, long latency (>2.8s), and high privacy. Thus, the Proposed Fed-LSH demonstrates a better balance among computational efficiency, query speed, and strong privacy protection.

**Table 2:** The time consumption of each calculation step of the client.

Phase	Encryption	Verification	Recovery
SM	md+td+2ml+4dl	td+dm+tm	tm+2ml

**Table 3:** The dataset in the experiments.

Dataset	Dimension d	Volume of data
CIFAR-10	32×32	60000
NCBI tomato gene data	1206-81465427	5156

**Table 4:** Performance comparison of Fed-LSH with other privacy-preserving methods.

Method	Computational cost	Query latency (s)	Privacy level
HElib (Lee et al., 2025; Tsuji and Oguchi, 2022)	Very High	>3.6	Very High
DP (Aziz et al., 2022)	Low	2.67	Low
SMPC (Nakagawa et al., 2022)	High	>2.8	High
Proposed Fed-LSH	Low	<0.5	High

## 5 Conclusion

Genomic data is on the scale of terabytes. To address the issue of rapid similarity matching of massive genomic data from multiple parties, we propose a federated LSH algorithm solution and framework. At the same time, we adopt cloud-assisted machine learning technology to alleviate the computational pressure on the Client side. To the best of our knowledge, there is currently no universal secure federated learning method applicable to all machine learning algorithms. Fully homomorphic encryption (FHE) is a feasible solution for achieving universal cloud-assisted machine learning, but the low computational efficiency of FHE makes FHE-based methods inapplicable to practical scenarios (Li et al., 2023). Differential privacy can also protect client data. By adding controllable noise, the original data cannot be identified, but the original data features are affected, which disrupts the characteristic signals on the genes (Wei et al., 2021). In the architecture proposed in this paper, the data on the Client side is all confidential, ensuring the privacy protection of Client data. Meanwhile, the Client can use optional edge computing and cloud-assisted machine learning to generate the HashTable of genomic data. With edge computing, the Client can directly use W to generate the HashTable. Alternatively, the cloud-assisted machine learning technology proposed above can be used to generate cloud-assisted batch HashTable for massive genomic data. The Data Server only stores the hash values of the genomic data, ensuring the privacy of the genomic data. At the same time, it can perform rapid similarity matching of massive genomic data from multiple parties.

The Fed-LSH framework proposed in this paper integrates federated learning, cloud-assisted machine learning, and LSH algorithm to solve the problem of rapid similarity matching of massive multi-party genomic data. The algorithm and framework have been tested for performance using tomato genome data and can also be applied to other genomic and whole-genome data, providing a standardized similarity matching tool for multi-institutional joint breeding. Through the deep coupling of federated learning and LSH, secure and efficient collaborative analysis of tomato genome data has been achieved.

Although we only conducted this test on the tomato genome, the Fed-LSH algorithm can be readily extended to fields such as crop gene matching (including rice, wheat, corn, etc.) and animal genetics. For PB-level genomic datasets, the algorithm demonstrates strong parallel computing potential and scalability, with performance significantly improved through parallel hash operations. The algorithm is particularly well-suited for high-performance computing and distributed computing environments. Cross-organizational genomic data sharing has raised critical ethical and privacy concerns. The proposed Fed-LSH framework supports compliance with GDPR and FAIR principles through fine-grained access control policies, comprehensive data usage audit logs, and dynamic consent mechanisms.

## 6 Challenges and prospects

However, the sharing of multi-party genetic data also faces many challenges. The differences in sequencing depth and annotation among different institutions can affect the consistency of gene hash values. Due to the long length and large volume of whole-genome data, creating a HashTable takes a considerable amount of time. GPU acceleration and distributed computing clusters can be adopted on Cloud Servers to speed up the calculation. Meanwhile, the standards for cross-border transmission of genetic data vary among different countries, and a more legal and compliant global privacy protection framework for cross-border transmission of genetic data needs to be designed. Combining blockchain technology with Fed-LSH technology can provide feasible solutions for data traceability and incentivizing multiple parties to provide genetic data.

## Data Availability

The datasets presenting in this study can be found here: CIFAR-10: https://www.cs.toronto.edu/~kriz/cifar.html; NCBI tomato gene data: https://www.ncbi.nlm.nih.gov/bioproject/?linkname=assembly_bioproject&from_uid=15321541.
